# Glial functions in the blood-brain communication at the circumventricular organs

**DOI:** 10.3389/fnins.2022.991779

**Published:** 2022-10-06

**Authors:** Seiji Miyata

**Affiliations:** Department of Applied Biology, Kyoto Institute of Technology, Kyoto, Japan

**Keywords:** brain, inflammation, microglia, macrophage, astrocyte, tanycyte, fluid homeostasis, body temperature

## Abstract

The circumventricular organs (CVOs) are located around the brain ventricles, lack a blood-brain barrier (BBB) and sense blood-derived molecules. This review discusses recent advances in the importance of CVO functions, especially glial cells transferring periphery inflammation signals to the brain. The CVOs show size-limited vascular permeability, allowing the passage of molecules with molecular weight <10,000. This indicates that the lack of an endothelial cell barrier does not mean the free movement of blood-derived molecules into the CVO parenchyma. Astrocytes and tanycytes constitute a dense barrier at the distal CVO subdivision, preventing the free diffusion of blood-derived molecules into neighboring brain regions. Tanycytes in the CVOs mediate communication between cerebrospinal fluid and brain parenchyma *via* transcytosis. Microglia and macrophages of the CVOs are essential for transmitting peripheral information to other brain regions *via* toll-like receptor 2 (TLR2). Inhibition of TLR2 signaling or depletion of microglia and macrophages in the brain eliminates TLR2-dependent inflammatory responses. In contrast to TLR2, astrocytes and tanycytes in the CVOs of the brain are crucial for initiating lipopolysaccharide (LPS)-induced inflammatory responses *via* TLR4. Depletion of microglia and macrophages augments LPS-induced fever and chronic sickness responses. Microglia and macrophages in the CVOs are continuously activated, even under normal physiological conditions, as they exhibit activated morphology and express the M1/M2 marker proteins. Moreover, the microglial proliferation occurs in various regions, such as the hypothalamus, medulla oblongata, and telencephalon, with a marked increase in the CVOs, due to low-dose LPS administration, and after high-dose LPS administration, proliferation is seen in most brain regions, except for the cerebral cortex and hippocampus. A transient increase in the microglial population is beneficial during LPS-induced inflammation for attenuating sickness response. Transient receptor potential receptor vanilloid 1 expressed in astrocytes and tanycytes of the CVOs is responsible for thermoregulation upon exposure to a warm environment less than 37°C. Alternatively, Na_*x*_ expressed in astrocytes and tanycytes of the CVOs is crucial for maintaining body fluid homeostasis. Thus, recent findings indicate that glial cells in the brain CVOs are essential for initiating neuroinflammatory responses and maintaining body fluid and thermal homeostasis.

## General introduction

The blood-brain barrier (BBB) was described more than 100 years ago with the observation that intravenous dye injection reached all organs but the brain (Ehrlich, 1885). The BBB is composed of a highly specialized brain endothelial cellular sheet that separates it from circulating blood and maintains brain homeostasis by tight and adherens junctions (Rubbin and Staddon, 1999; Tsukita et al., 2001; Hawkins and Davis, 2005; Zlokovic, 2011). The BBB does not preclude blood-to-brain communication as, *via* transporters and transcytosis, it permits the exchange of molecules such as peptides, proteins, vitamins, hormones, amino acids, nucleotides, fatty acids, and other compounds (Miller and Leslie, 1994; Barar et al., 2016; Yang et al., 2020).

**FIGURE 1:**
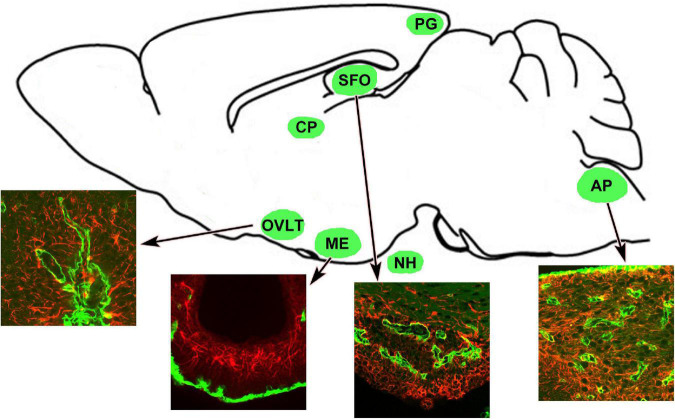
Schematic representation revealing CVOs locations in the adult rodent brain. Insets indicate that the CVOs are characterized by dense and thick laminin^+^ (green) capillaries and dense GFAP^+^ (red) astrocytic and tanycytic networks. Scale bar = 50 μm. AP, area postrema; CP, choroid plexus; GFAP, glial fibrillar acidic protein; ME, median eminence; NH, neurohypophysis; OVLT, organum vasculosum of the lamina terminalis; PG, pineal gland; SFO, subfornical organ. The illustration is rearranged with permission from Elsevier B.V. (Takagi et al., 2017).

The circumventricular organs (CVOs) are located around the brain ventricles and lack the BBB (Hofer, 1987; Johnson and Gross, 1993; Ganong, 2000; Fry et al., 2007; Sisó et al., 2010a,b; Mimee et al., 2013; Miyata, 2015, 2017), thereby allowing blood-derived molecules to be sensed, and the release of brain-synthesized neuropeptides into circulation (Miyata and Morita, 2011; Morita and Miyata, 2012; Morita et al., 2013, 2016; Miyata, 2015, 2017). Therefore, the CVOs are referred to as the “windows of the brain” (Gross and Weindl, 1987). The CVOs comprise the organum vasculosum of the lamina terminalis (OVLT), subfornical organ (SFO), median eminence (ME), area postrema (AP), neurohypophysis (NH), pineal gland, and choroid plexus shown in Figure 1 (Takagi et al., 2017). The CVOs are divided into two groups based on their primary role, sensory and secretory. The sensory CVOs include the OVLT, SFO, and AP, and they monitor ions, osmolality, pH, lipophobic amino acids, and neuropeptides in blood circulation *via* a wide variety of receptors (Sisó et al., 2010a,b; Ferguson, 2014). In addition, they integrate and transmit blood-derived information to neighboring brain regions to control inflammation and body fluid and thermal homeostasis (Roth et al., 2004; Sisó et al., 2010a,b; Miyata, 2015). The secretory CVOs, consisting of the ME, NH, and pineal glands release various neuropeptides or melatonin (Miyata and Hatton, 2002; Miyata, 2017). The innermost layer of the meninges forms vascularized invaginations, called choroid plexus, in some parts of the ventricles to produce the CSF. Our previous review describes detailed functions of the secretory CVOs (Miyata, 2017).

The lack of the typical endothelial BBB does not mean that the fenestrate capillary in the CVOs allows free entry of blood-derived molecules to the parenchyma since only low blood-derived low-molecular-weight (LMW) molecules can pass through the fenestrate capillary (Morita and Miyata, 2012; Miyata, 2015; Morita et al., 2016). Moreover, blood-derived LMW molecules cannot diffuse into neighboring brain regions by dense walls constituted by astrocytes and tanycytes at distal CVO subdivisions. Tanycytes are specialized ependymal or glial cells found in the ventricular system and CVOs of the brain and possess long cellular processes extending deep into the brain parenchyma (Rodríguez et al., 2005, 2019). Tanycytes are classified as reminiscent radial glia owing to their highly polarized morphology and neural stem cell niche (Rodríguez et al., 2005; Furube et al., 2020).

The CVOs are essential brain regions for neuroinflammatory response initiation, for circulating pathogen-associated molecular patterns (PAMPs) and cytokines to induce faster transcriptional activation of genes encoding for pro-inflammatory molecules (Quan et al., 1997; Rummel et al., 2005; Yoshida et al., 2016). The CVOs are thought to function in pathological conditions such as sepsis, stress, the neuronal invasion of parasites, autoimmune encephalitis, and systemic amyloidosis (Sisó et al., 2010a,b; Kristensson et al., 2013), as they are involved in most autonomic and endocrine regulatory pathways and lack the BBB. The CVOs are also essential to the brain region for sensing thermal and osmotic signals and integrating their information to initiate proper physiological homeostatic responses (Sladek and Johnson, 2013; Noda and Hiyama, 2015; Park et al., 2020; Ilanges et al., 2022).

This review introduces advances in understanding the involvement of CVO glial cells in brain-to-periphery communication at the blood-brain interface. In this review, the following aspects of the CVOs are discussed: (1) Fundamental properties of fenestrated capillaries with size-limited permeability, glial barriers, and tanycytic transcytosis; (2) Significance of pattern recognition receptors such as toll-like receptors 2 (TLR2), TLR4, and glial cell inflammatory signal transduction from the periphery to the brain, and (3) Maintenance of body fluid and thermal homeostasis by glial Na_*x*_ channel and transient receptor potential vanilloid 1 (TRPV1), respectively.

### Size-limited vascular permeability at fenestrated capillaries

The BBB is important for normal brain physiology and protects neuronal tissues by limiting the ingress of blood-derived bioactive and toxic hydrophilic molecules. Disturbance of the BBB leads to the accumulation of these molecules within the parenchyma causing severe brain damage (Zlokovic, 2011; Obermeier et al., 2013). Mice deficient in tight junction proteins such as claudin-5 or ZO-1 die due to blood-derived toxic molecules accessing the brain (Morita et al., 1999; Furuse et al., 2001, 2002; Katsuno et al., 2008). However, CVO vasculature lacks the typical BBB, and possesses fenestrated features unlike those in other brain regions (Miyata and Morita, 2011; Morita and Miyata, 2012; Morita et al., 2013, 2016; Miyata, 2015, 2017). For example, levels of glutamate, an excitatory transmitter, in plasma and brain is in the range of 50–100 and 0.5–2 μmol/l, respectively (Hawkins, 2009). Even after oral ingestion of large quantities of glutamate, only small changes in glutamate plasma concentration occur (Stegink et al., 1982). The subcutaneous administration of high-dose monosodium glutamate increases fivefold increase of glutamate levels in the SFO and AP (Price et al., 1981). The administration of monosodium glutamate also induces neuronal loss in the ME and arcuate nucleus (Arc) of adult (Yulyaningsih et al., 2017; Heiss et al., 2022) and the CVOs of neonatal rodents (Lemkey-Johnston and Reynolds, 1974). Many studies demonstrate the free diffusion of LMW molecules into the CVOs parenchyma: fluorescein (MM = 332), fluorescein isothiocyanate (FITC; MM = 390), Evans blue (MM = 961), and Dextran 3,000 (MW = 3,000) (Willis et al., 2007; Morita and Miyata, 2012; Morita et al., 2013, 2016; Nishikawa et al., 2017). The molecular weight of these tracer molecules is within the range of bioactive neuropeptides, amino acids, or amines; e.g., adenohypophyseal hormone-releasing hormones such as thyrotropin-releasing hormone (minimum size at MW = 362.4) to growth-hormone-releasing hormone (maximum size at MW = 5040.4). In contrast to LMW molecules, blood-derived high-molecular-weight (HMW) molecules, more than MW 10,000, do not freely enter the CVOs parenchyma (Willis et al., 2007; Morita and Miyata, 2012; Morita et al., 2016). LMW soluble molecules (<3 nm molecular radius) are presumed to move passively through endothelial intercellular space in BBB-lacking capillaries (Mehta and Malik, 2006; Komarova and Malik, 2010). Thus, the CVOs endothelial intercellular space exhibits a size-limited vascular permeability lower than approximately MW 10,000 (Miyata, 2015, 2017). However, it is often misunderstood that the fenestrated capillaries of the CVOs allow molecules of any size to enter the parenchyma.

High-molecular-weight molecules cannot pass freely through fenestrate capillaries in the CVOs. HMW molecules such as hormones and cytokines reach the brain parenchyma beyond the BBB *via* transcytosis (Gonçalves and De Felice, 2021). Recently, more than a thousand proteins, including various cytokines and growth factors, readily permeated brain parenchyma with the transcytosis system by BBB-specific transcriptional programs (Yang et al., 2020). Autoradiographic and fluorescent microscopic techniques show heterogenous transcytosis of blood proteins within the brain, especially prominent at the basolateral hypothalamus, including the ME, Arc, and ventromedial hypothalamic nucleus (Yang et al., 2020). Blood-derived leptin activates leptin receptors in tanycytes of the ME and is transported along the tanycytic cellular process and secreted into the CSF (Balland et al., 2014). Blood-derived horseradish peroxidase extensively accumulates between the inner and outer BM in the NH and ME, but it also reaches the brain parenchyma, possibly *via* mannose receptor-mediated transcellular routes (Ellinger and Fuchs, 2010; Rodríguez et al., 2010).

### Astrocytic and tanycytic barrier instead of endothelial blood-brain barrier

**FIGURE 2:**
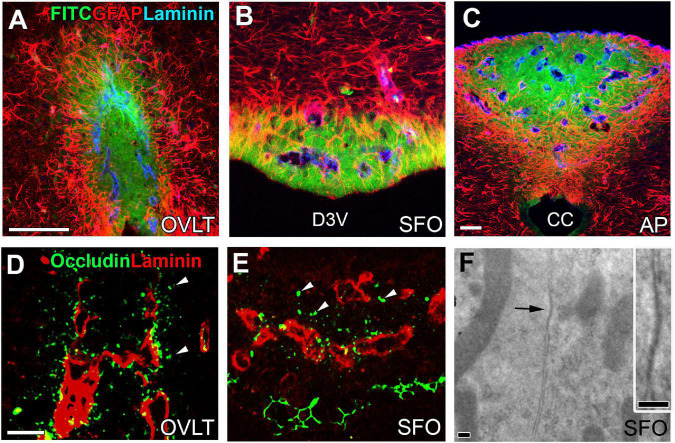
Vascular permeability and tight junctions in the CVOs of the adult mouse brain. Strong fluorescence of blood-derived fluorescein isothiocyanate is seen at the central subdivision of the sensory CVOs, but fluorescence is weak at distal subdivisions and no fluorescence is seen in the neighboring brain region beyond dense astrocytic and tanycytic barriers **(A–C)**. Tight junction protein occludin is seen at parenchyma close to fenestrated capillaries and tanycytic layers **(D,E)**. An electron microscopic image reveals the tight junction-like structure between the plasma membrane of neighboring cells making complete contact **(F)**. Scale bar = 50 μm **(A,C,D)** and 100 nm **(F)**. AP, area postrema; CC, central canal; D3V, dorsal 3rd ventricle; FITC, fluorescein isothiocyanate; GFAP, glial fibrillar acidic protein; OVLT, organum vasculosum of the lamina terminalis; SFO, subfornical organ. Photographs are rearranged with permission from Springer Nature (Morita et al., 2016).

The basement membrane of fenestrated capillaries is permeable to blood-derived LMW molecules, which easily reach the CVOs parenchyma; however, they do not diffuse out of the CVO, as shown in Figures 2A–C (Morita et al., 2016). The capillaries of central subdivisions in the sensory CVOs possess a higher vascular permeability than those of distal subdivisions due to the lack of tight-junction protein expression in the endothelial cells (Miyata, 2015; Morita et al., 2016). Astrocytes in the distal regions of the sensory CVOs become hypertrophic and their density is remarkably high (Morita et al., 2016). Tanycytes in the OVLT and SFO, but not in the AP, extend cellular processes into the surrounding neuronal somata and fenestrated capillaries, connecting tightly (Noda and Hiyama, 2015; Prager-Khoutorsky and Bourque, 2015; Morita et al., 2016). Astrocytes and tanycytes often express several kinds of tight junction proteins (Figures 2D,E; Morita et al., 2016), and electron microscopy reveals junctions between intimately apposed astrocytic or tanycytic cellular membranes (Figure 2F; Morita et al., 2016). Astrocytes are reported to express ZO-1 and occludin in the periphery of infarct areas during the stroke recovery process to prevent ingress of neurotoxic molecules (Yang et al., 2007, 2013). Thus, the astrocytes and tanycytes, densely located at distal CVO subdivisions, are an alternative to the endothelial BBB and prevent blood-derived LMW molecules from diffusing into neighboring brain regions.

### Inability of blood-derived high-molecular-weight molecules to cross fenestrated capillaries into the parenchyma

In contrast to the high vascular permeability of blood-derived LMW molecules in the CVOs fenestrated capillaries, HMW molecules, MW > 10,000, can cross the outer basement membrane but do not diffuse into parenchyma past the inner basement membrane and accumulate between these membranes (Morita and Miyata, 2012; Morita et al., 2016; Nishikawa et al., 2017). Continuous capillaries in the typical BBB-containing brain vasculature possess a narrow perivascular space surrounded by inner and outer basement membrane generally fused, whereas fenestrate capillaries in the CVO have a large and complex perivascular space (Dellmann, 1998; Morita et al., 2016). Increased collagen type I expression is detected on the inner basement membrane compared with the outer basement membrane, whereas laminin expression is increased in the outer basement membrane compared with the inner (Morita et al., 2016; Nishikawa et al., 2017). These features are also seen in the kidney glomerulus, the glomerular basement membrane contributing to the size-limited kidney filtration barrier that separates proteins and LMW molecules such as glucose and urea (Chen and Miner, 2012; Miner, 2012). Mutations in either laminin or collagen type IV result in proteinuria, often leading to nephrotic syndrome and renal failure (Chen and Miner, 2012). Thus, like typical BBB-containing capillaries, fenestrated capillaries of the CVOs prevent parenchyma access to HMW molecules beyond the inner basement membrane. Blood-derived albumin in the brain parenchyma causes neurodegeneration (Mallmann et al., 2022), demonstrating the neurotoxicity of blood-derived HMW molecules. However, this evidence does not proscribe all HMW molecule access to the parenchyma, as recent studies found that active transport of blood-derived HMW molecules into the brain parenchyma occurs by ligand-specific receptor-mediated and non-specific caveolar transcytosis (Ellinger and Fuchs, 2010; Yang et al., 2020; Gonçalves and De Felice, 2021).

### Tanycytic transcytosis mediated cerebrospinal fluid-brain communication in the circumventricular organs

Tanycytes are specialized ependymal cells interfacing with the CSF and have a long basal process that terminates at the capillary (Horstmann, 1954; Rodríguez et al., 2005). Ependymal cells in the Arc and ME are the best-known tanycytes and have a single, long cellular process projecting to discrete hypothalamic parenchyma and fenestrated capillaries (Rodríguez et al., 1979, 2005). Tanycytes also exist at the ventricular borders of the OVLT and SFO (Mullier et al., 2010; Langlet et al., 2013). A honeycomb distribution pattern of tight junction proteins is observed at tanycytes around the CVO proximal cell bodies, possibly to prevent the diffusion of blood-derived molecules into the CSF (Mullier et al., 2010; Langlet et al., 2013). Hypothalamic tanycytes also reduce the release into circulation of thyrotropin-releasing hormone by activating the tanycytic receptor for thyrotropin-releasing hormone (Müller-Fielitz et al., 2017). However, circulating leptin is recognized by leptin receptors on tanycytes in the ME, then extracellular signal-regulated kinase signaling of tanycytes promotes leptin transport, activating neurons in the mediobasal hypothalamus (Balland et al., 2014). Tanycytes participate in the release of gonadotropin-releasing hormone by the spatial relationship between the tanycytes and gonadotropin-releasing hormone-secreting terminals (Prevot et al., 2010; Bolborea and Dale, 2013). Hypothalamic tanycytes also reduce the release of thyrotropin-releasing hormone into the circulation by activating the tanycytic receptor for thyrotropin-releasing hormone (Müller-Fielitz et al., 2017). Active transcytosis occurs at tanycytes in the CVOs of the adult mouse brain, as in the Arc (Okamoto et al., 2022). The CVOs can, by tanycytic transcytosis, transport bioactive molecules between the CSF and CVO, suggesting that the long cellular processes of tanycytes act as communication routes between the CSF and brain parenchyma.

### Circumventricular organ tanycytes are neural stem cells

Tanycytes in the ME and Arc are shown to be a neurogenic niche in adult mammals (Lee et al., 2012; Robins et al., 2013). Tanycytes in the spinal cord are NSCs that, remarkably, proliferate and differentiate into astrocytes and oligodendrocytes upon spinal injury (Meletis et al., 2008). Recently we have found two types of NSCs in the CVOs; astrocyte- and tanycyte-like NSCs (Hourai and Miyata, 2013; Furube et al., 2015, 2020; Nambu et al., 2021a,b). Radial glial cells in the ventricle zone are NSCs that, during cortical development, produce new neurons and glial cells (Ohtaka-Maruyama and Okado, 2015). Tanycytes in the adult mammalian brain are NSC precursors, ultimately derived from radial glia during development (Goodman and Hajihosseini, 2015). The CSF provides important functions to support brain homeostasis, morphogenesis, and proliferation during development (Johansson, 2014). In adults, the CSF actively synthesizes and secretes factors that promote the proliferation of quiescent and transit-amplifying NSCs in the subventricular zone (Silva-Vargas et al., 2016). Aging speed is controlled primarily by hypothalamic NSCs *via* levels of exosomal miRNAs in the CSF (Zhang et al., 2017). Moreover, continuous angiogenesis occurs in the CVOs of adult mice, possibly reconstructing neurons, glia, and fenestrated capillaries (Morita et al., 2013, 2015; Furube et al., 2014; Asami et al., 2020). Thus, tanycytes, throughout the adult mammalian brain, are accepted as NSCs, with their proliferation and differentiation potentially regulated by factors from the CSF and fenestrated capillaries, depending on body conditions.

### The circumventricular organs are a possible communication route between the periphery and brain

Peripheral immune response transmits information to the brain to induce physiological and behavioral responses, but communication between the peripheral immune signal molecules or PAMPs and brain parenchyma is not well understood. Hypotheses proposed as to how peripheral inflammatory signals and PAMPs in circulation are transferred to the brain parenchyma to stimulate associated neural circuits include; 1) cytokine-dependent prostaglandin E_2_ (PGE_2_) synthesis and release at endothelial cells of brain vasculature, 2) activation of parenchymal cells in the CVOs by blood-derived molecules across fenestrated capillaries, 3) activation of peripheral nerves by cytokines and PGE_2_ (Blomqvist and Engblom, 2018). However, it is difficult to conclude that a single route transmits peripheral inflammatory information to the brain, as neuroinflammation is associated with various physiological changes such as body temperature, locomotor activity, food and water intake, and metabolism (Tesoriero et al., 2018). Therefore, several routes may work in conjunction to regulate complex physiological inflammation responses.

This section focuses on glial cells in CVOs with a specialized niche for the blood-brain interface to transport information from the periphery to the brain. The CVOs express a range of inflammation-associated receptors for cytokines, PAMPs, neuropeptides, and PGE_2_ (McKinley et al., 2003; Roth et al., 2004; Sisó et al., 2010a,b; Miyata, 2015). In comparison to other brain regions, peripheral inflammatory stimulation in the CVOs causes faster and stronger transcriptional activation of a vast number of proinflammatory molecules; such as nuclear factor-κ B (NF-κB; Quan et al., 1997; Laflamme et al., 1999; Chakravarty and Herkenham, 2005; Muneoka et al., 2019; Murayama et al., 2019), the signal transducer and activator of transcription factor 3 (STAT3; Rummel et al., 2004, 2005; Nakano et al., 2015; Yoshida et al., 2016), and Fos (Konsman and Dantzer, 1999; Muneoka et al., 2019; Murayama et al., 2019; Takagi et al., 2020). Microsomal prostaglandin E synthase-1, an inducible catalytic enzyme for converting PGH_2_ to PGE_2_, is highly expressed in autonomic relay structures such as the CVOs, preoptic area (POA), Arc, and paraventricular nucleus (Eskilsson et al., 2014). Importantly, microsomal prostaglandin E synthase-1 expression is observed in endothelial cells, pericytes, and neurons of the CVOs. Sickness responses induced by interleukin-1β (IL-1β) are not derived from cerebral parenchymal vasculature but depend on a relatively small number of fenestrated capillaries located in the CVOs (Knoll et al., 2017). Strong expression of receptors against cytokines such as IL-6, IL-1β, and tumor necrosis factor-α (TNF-α) is also demonstrated in the CVOs (Ericsson et al., 1995; Vallieres and Rivest, 1997; Nadeau and Rivest, 2000; Roth et al., 2009). IL-6-deficient mice do not exhibit fever generation in response to peripheral administration of lipopolysaccharide (LPS) in contrast to IL-1β and TNF-α-deficient animals (Chai et al., 1996). Also, remarkably high expression of TLR2 and TLR4 mRNA occurs in the CVOs (Laflamme et al., 2001; Laflamme and Rivest, 2001).

The AP forms the dorsal vagal complex with the solitary nucleus (Sol) and plays a role in the emetic reflex, immune responses, cardiovascular regulation, and energy homeostasis (Rogers et al., 1996; Price et al., 2008). The Arc and AP are the most critical brain regions for circulating factors involved in regulating food intake and energy homeostasis (McKinley et al., 2003; Price et al., 2008; Riediger, 2012). The AP is known to be implicated in nausea and vomiting to at least visceral stimulation (Miller and Leslie, 1994). Blood levels of growth/differentiation factor 15 are increased in sickness-associated events such as bacterial and viral infection, LPS, lithium chloride, and pregnancy (Tsai et al., 2018). Growth/differentiation factor 15 receptor-expressing excitatory neurons in the AP mediate aversion responses to visceral LPS and lithium chloride (Zhang C. et al., 2020). TNF-α stimulates neurons in the AP to elicit sympathetic excitation and blood pressure increases (Korim et al., 2019). Thus, the AP is chiefly concerned with inflammation-induced sickness responses, such as decreased food intake, nausea, and hypertension.

The OVLT is located close to the POA that regulates body temperature, and sizeable electrolytic lesion to the anteroventral 3rd ventricle (AV3V) region, including the OVLT and SFO, attenuates fever after peripheral administration of LPS (Blatteis et al., 1983; Stitt, 1985). The quantitative autoradiography of [^3^H] PGE_2_ shows the highest binding density in the AV3V wall surrounding the OVLT (Matsumura et al., 1990). Subsequent studies indicate that fever-generation abnormalities come from side effects due to body fluid deficiency, electrolyte, and cardiovascular homeostasis (Romanovsky et al., 2003). In contrast to the OVLT, an electrical lesion to the SFO prevents LPS-induced fever (Takahashi et al., 1997). Direct injection of IL-1β into the SFO increases body temperature and salt intake (Cerqueira et al., 2016).

When the SARS-CoV-2 virus reaches the brain, the subsequent neurological damage results in dysfunctions of long-term body condition (Graham et al., 2021). SARS-CoV-2 binds to angiotensin-converting enzyme 2 (ACE2) on brain endothelial cells and induces inflammatory responses and activation of microglia and astrocytes to disrupt the integrity of the BBB (Buzhdygan et al., 2020; Ong et al., 2022). Infection of SARS-CoV-2 to brain neurons also induces the release of inflammatory molecules and ROS and free radical formation (Sindona et al., 2021). ACE2 is highly expressed in the CVOs and brain regions connecting to the CVOs, such as paraventricular nucleus and Sol (Doobay et al., 2007). The CVOs are essential brain regions to control angiotensin-dependent regulation of osmotic thirst, the release of arginine-vasopressin (AVP) and oxytocin (OXT) release, and blood pressure (Ferguson, 2014). In addition to ACE2, SARS-CoV-2 virus interacts directly with TLR4 to cause an immune response and increase cell surface expression of ACE2 (Bhattacharya et al., 2020; Aboudounya and Heads, 2021; Zhao et al., 2021). TLR4 protein is highly expressed in astrocytes and tanycytes in the CVOs of adult mice (Nakano et al., 2015; Muneoka et al., 2019). These facts suggest that the CVOs are possibly the primary route of entry of SARS-CoV-2 virus into the brain.

In conjunction with viral and bacterial infection, the CVOs are also involved with parasitic infection. African trypanosomes first infect the ME fenestrated capillaries, then move, by rotating flagella and protease secretion, into adjacent hypothalamic regions such as the Arc and suprachiasmatic nucleus (Grab et al., 2009; Huet et al., 2009; Kristensson et al., 2013; Bargul et al., 2016; Bentivoglio et al., 2018).

### Toll-like receptor 2 in microglia and macrophages of the circumventricular organs

Toll-like receptors recognize PAMPs, which play crucial roles in early innate recognition and host inflammatory responses against invading disease-causing microbes (Akira et al., 2001; Gay et al., 2014). Studies show that expression of TLR2 and TLR4 is remarkably higher in the CVOs of the adult brains than in other brain regions (Nakano et al., 2015; Muneoka et al., 2019; Murayama et al., 2019). Circulating LPS has limited access to the brain parenchyma in non-CVOs region due to the BBB, but it can reach the parenchymal in the CVOs possibly *via* the lipoprotein transport system (Vargas-Caraveo et al., 2017). Moreover, a TLR2 ligand zymosan also reaches the parenchymal in the CVOs, although the transport system is unclear (Takagi et al., 2020). These shreds of evidence suggest peripheral endotoxins can activate parenchymal TLR2 and TLR4 in the CVOs. Moreover, TLR2 and TLR4 have crucial functions in modulating inflammatory responses of the brain *via* endogenous ligands such as heat-shock proteins, high mobility group box 1, hyaluronic acids, and fibronectin (Wang et al., 2013; Li et al., 2021). However, the specialized spatial niches suggest that TLR2 and TLR4 ligands, derived from pathogens in the blood, activate TLR2 and TLR4 in the CVOs. This indicates that the CVOs can directly detect PAMPs, and integrate and relay information to deep brain regions, and initiate brain neuroinflammation.

**FIGURE 3:**
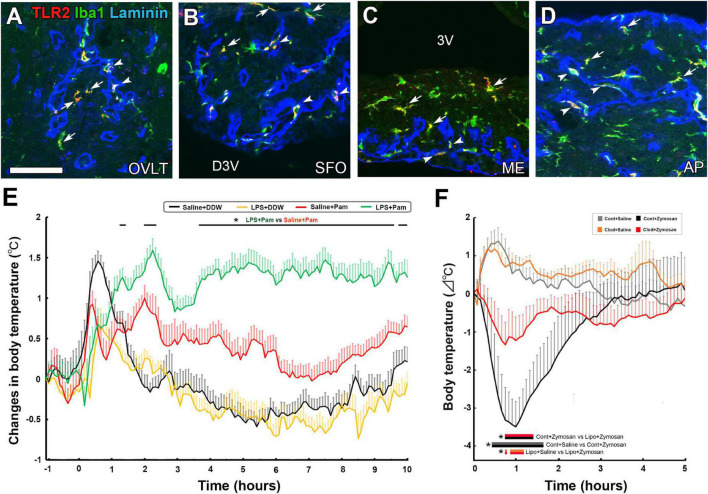
The expression of TLR2 at microglia and macrophages in the CVOs and TLR2 agonist-induced fever and hypothermia in the adult mouse. Triple labeled immunohistochemistry shows prominent TLR2 expression at Iba1^+^ microglia (arrows) in parenchyma and Iba1^+^ macrophages (arrowheads) in laminin^+^ capillary of the CVOs **(A–D)**. LPS pretreatment promotes fever generation induced by a LMW TLR2 agonist Pam3CSK4 **(E)**. Depletion of microglia and macrophages with clodronate liposomes attenuates hypothermia induced by the HMW TLR2 agonist zymosan **(E)**. Scale bar = 50 μm. 3V, 3rd ventricle; AP, area postrema; D3V, dorsal 3rd ventricle; ME, median eminence; OVLT, organum vasculosum of the lamina terminalis; SFO, subfornical organ; TLR2, toll-like receptor 2. *p* < 0.05 by an ANOVA with Tukey’s *post-hoc* test. Photographs are rearranged with permission from Elsevier B.V. [**(A–E)** Murayama et al., 2019; **(F)** Takagi et al., 2020].

Among TLR family members, TLR2 recognizes a variety of TLR2 molecules from viruses, fungi, bacteria, and parasites by forming a heterodimer with TLR1 or TLR6 (Zähringer et al., 2008; Oliveira-Nascimento et al., 2012). TLR2 heterodimers generally initiate a MyD88-dependent intracellular signaling pathway so that NF-κB is activated and translocated to the nucleus to modulate gene transcription of cytokines (Kawai and Akira, 2010). The activation of TLR2 in immune cells by lipoproteins is a significant factor in Gram-positive sepsis (Takeuchi et al., 2000; Hashimoto et al., 2006). In the CVOs, TLR2 mRNA expression was higher than in other brain regions (Laflamme et al., 2001), and TLR2-expressing cells are identified as microglia and macrophages in the CVOs (Figures 3A–D; Murayama et al., 2019). The systemic administration of macrophage-activating lipopeptide-2 induces the activation of STAT3 in a subpopulation of parenchyma cells in the CVOs, together with fever generation (Knorr et al., 2008). Peripheral administration of the LMW TLR2 agonist Pam3CSK4 caused NF-κB activation in microglia and macrophages and Fos expression in astrocytes, tanycytes, and neurons in the CVOs (Murayama et al., 2019). Peripheral and intracerebroventricular (i.c.v.) injection of a TLR2 ligand, Pam3CSK4, leads to sickness responses, including anorexia, hypoactivity, and fever, depending on NF-κB and PGE_2_ (Jin et al., 2016; Murayama et al., 2019). A robust and temporal elevation of the TLR2 mRNA expression occurs in the CVOs after the systemic injection of a TLR4 agonist LPS (Laflamme and Rivest, 2001; Rivest, 2003, 2009), indicating crosstalk between TLR4 and TLR2. Pam3CSK4-induced fever is augmented by pretreatment of LPS (Figure 3E; Murayama et al., 2019). The expression of TLR2 is reported in both perivascular macrophages and parenchyma microglia of the CVOs (Murayama et al., 2019; Takagi et al., 2020). The CVO perivascular structure is largely different from that of the general brain; Firstly, a broad perivascular space is present between the inner and outer basement membranes in the CVOs, whereas brain capillaries generally fuse, thus lacking perivascular space (Miyata, 2015, 2017). Secondly, blood-derived HMW molecules accumulate in the perivascular space (Morita et al., 2016; Miyata, 2017; Nishikawa et al., 2017). Perivascular macrophages possess crucial functions such as maintaining vascular permeability, pathogen phagocytosis, antigen representation, and limiting endothelial cell production of PGE_2_ to avoid unnecessary inflammation (Lapenna et al., 2018). IL-1β signaling in fenestrated capillaries of the CVOs is sufficient to trigger sickness responses in mice (Knoll et al., 2017).

The depletion of microglia and macrophages with clodronate liposomes (Clod-Lips) has been shown to attenuate fever caused by the i.c.v. administration of Pam3CSK (Jin et al., 2016). I.c.v injection of Clod-Lips reduced zymosan-induced Fos expression in astrocytes, tanycytes, and neurons in the CVOs, while a Clod-Lips injection attenuated zymosan-induced hypothermia (Figure 3F; Takagi et al., 2020). Thus, microglia and macrophages are primary cells in initiating faster and stronger transcriptional activation of the CVOs, at least partially *via* direct activation of TLR2 and transmitting peripheral information to neurons in the CVOs to cause sickness responses in TLT2-dependent neuroinflammation.

### TLR4 in astrocytes and tanycytes in the circumventricular organs

**FIGURE 4:**
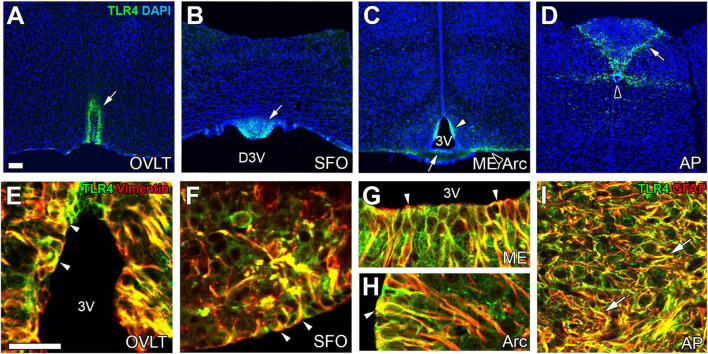
The expression of TLR4 at astrocytes/tanycytes of the adult mouse brain. Immunohistochemistry shows robust and specific TLR4 expression in the CVOs (open arrows), Arc (open arrowhead), and CC (solid arrowhead) **(A–D)**. Double-labeling immunohistochemistry reveals that TLR4 is expressed at vimentin^+^ tanycytes (arrowheads) and GFAP^+^ astrocytes (arrows) **(E–I)**. Scale bars = 50 μm. 3V, 3rd ventricle; AP, area postrema; Arc, arcuate nucleus; DAPI, 4′,6-diamidino-2-phenylindole; D3V, dorsal 3rd ventricle; GFAP, glial fibrillar acidic protein; ME, median eminence; OVLT, organum vasculosum of the lamina terminalis; SFO, subfornical organ; TLR4, toll-like receptor 4. Photographs are rearranged with permission from Elsevier B.V. (Muneoka et al., 2019).

The expression of TLR4 mRNA is higher in the CVOs than in other brain regions (Laflamme and Rivest, 2001; Chakravarty and Herkenham, 2005). TLR4 protein is expressed in astrocytes and tanycytes in the CVOs of adult mice (Figure 4; Muneoka et al., 2019). The peripheral administration of LPS upregulates mRNA levels of CD14, which helps bind TLR4 to LPS-binding protein in the CVOs (Lacroix et al., 1998; Nadeau and Rivest, 2000). Peripheral administration of LPS activates STAT3 (Rummel et al., 2005; Yoshida et al., 2016) and NF-κB signaling (Laflamme et al., 1999; Muneoka et al., 2019) in astrocytes and tanycytes of the CVOs. The i.c.v. injection of LPS also causes STAT3 signaling activation in astrocytes and tanycytes of the CVOs (Nakano et al., 2015; Yoshida et al., 2016). There is no apparent difference in the activation of NF-κB and STAT3 between astrocytes and tanycytes after peripheral administration of LPS (Nakano et al., 2015; Muneoka et al., 2019). In TRM8 KO mice, (Shiraki et al., 2021; Kasuga et al., 2022) i.c.v. injection of an LPS antagonist LPS-RS attenuates NF-κB signaling in the astrocytes and tanycytes of the CVOs after peripheral administration of LPS (Muneoka et al., 2019). Thus, the cellular phenotype for faster and more robust activation of transcriptional activation is largely different between TLR2 and TLR4 in the CVOs; TLR2 in microglia and macrophages, and TLR4 in astrocytes and tanycytes. Thus, astrocytes are important in initiating neuroinflammation in the CVOs at least partially by TLR4 and transmitting peripheral information to neurons in the CVOs.

The microglial and macrophage depletion by Clod-Lips enhanced the fever induced by peripheral administration of LPS (Serrats et al., 2010). The depletion of microglia and macrophages with the antagonist to the colony-stimulating factor-1 receptor and transgenic mouse fractalkine receptor does not attenuate LPS-induced sickness responses such as body-weight decrease, locomotor activity, and exacerbates voluntary wheel running (Vichaya et al., 2020). Moreover, it is shown that the depletion of microglia and macrophages does not prevent proinflammatory cytokine expression and exacerbates some cytokines in LPS-induced inflammation (Vichaya et al., 2020). Astrocytes have emerged as key contributors to the innate immune response of the brain to infections, neurodegenerative disorders, and injuries (Bsibsi et al., 2006; Farina et al., 2007; Li et al., 2021). These results suggest that TLR4-dependent sickness responses in acute inflammation are caused independently of microglia activation.

In contrast, we cannot deny the possibility that microglia participate in acute and chronic neuroinflammation during LPS-induced inflammation. For example, microglia inhibitor minocycline promotes recovery from sickness behaviors and reduces mRNA expression of proinflammatory cytokines upon LPS stimulation without changing plasma IL-1β levels (Henry et al., 2008). Minocycline treatment decreases STAT3 activation in astrocytes and tanycytes of the OVLT and AP, but not SFO, upon peripheral administration of LPS (Nakano et al., 2015). Central injection of TLR4 agonists induces morphological activation of microglia (Vargas-Caraveo et al., 2020). The treatment of LPS antagonist RS-LPS results in a markedly elongated morphology of microglia in the three CVOs, a shape generally seen in severe brain injury and infection (Vargas-Caraveo et al., 2020). Microglial proliferation in the brain is ubiquitous in chronic inflammation and exerts beneficial effects by attenuating sickness responses under LPS-induced chronic inflammation (Michels et al., 2019; Torii et al., 2022). Thus, the functional significance of microglia and macrophages in TLR4-dependent inflammation is largely different from TLR2, although they may work in conjunction with astrocytes during neuroinflammation.

### Unique properties of microglia and macrophages in the circumventricular organs

**FIGURE 5:**
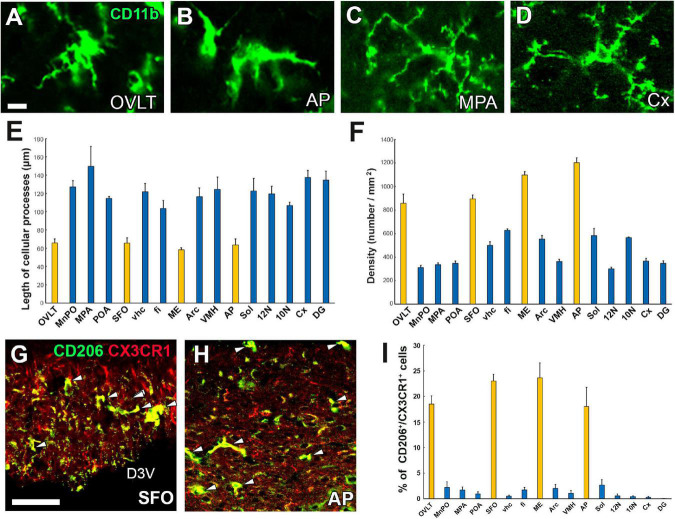
Activated state of microglia in the CVOs even under physiologically normal conditions. Microglia morphology in the CVOs, such as the OVLT and AP, is amoeboid, whereas in the medial preoptic area and cerebral cortex, it is dendritic **(A–D)**. Quantitative analysis reveals shorter microglial cellular processes in the CVOs than other brain regions **(E)**. The density of the CVOs microglia is significantly higher than that of other brain regions **(F)**. Double-labeling immunohistochemistry shows that CX3CR1^+^ microglia express an M2 marker protein CD206 (arrowheads) in the SFO and AP **(G,H)**. Quantitative analysis shows the percentage of CD206 expression in CX3CR1^+^ microglia is higher in the CVOs than that of other brain regions **(I)**. 10N, dorsal motor nucleus of vagus nerve; 12N, hypoglossal nucleus; AP, area postrema; Cx, cerebral cortex; D3V, dorsal 3rd ventricle; DG, dentate gyrus; fi, fimbria; ME, median eminence; MnPO, median preoptic area; MPA, medial preoptic area; OVLT, organum vasculosum of the lamina terminalis; SFO, subfornical organ; Sol, solitary nucleus; vhc, ventral hippocampal commissure; VMH, ventromedial hypothalamic nucleus. Scale bars = 10 **(A)** and 50 μm **(G)**. Scale bars = 50 μm. Yellow and blue bars indicate the CVOs and non-CVO regions, respectively. Photographs are rearranged with permission from Elsevier B.V. (Takagi et al., 2017).

Microglia are resident macrophage-like cells of mesoderm origin and are distinct from other types of neuroglia in the brain (Kettenmann et al., 2011). Although microglia are considered quiescent under normal physiological conditions, activated only upon an immune challenge or mechanical damage, recent evidence indicates that they control neuronal function and homeostasis without requiring these stimuli (Frick et al., 2013). For example, microglia are involved in synaptic pruning and the generation of neurons and oligodendrocytes (Paolicelli et al., 2011). Microglia exhibit a ramified morphology, a so-called “resting state” under physiologically normal conditions, but rapidly transform to amoeboid after infection of pathogens, toxins, mechanical injury, or radiation, together with dramatically increased proinflammatory cytokine gene expression and promotion of phagocytosis for dying cells or pathogens (Hanisch and Kettenmann, 2007; Colton, 2009). In the CVOs, surprisingly, microglia adopt the amoeboid form with fewer branched cellular processes even under normal conditions, whereas those in other brain regions exhibit the ramified form with well-branched and dendritic cellular processes (Figures 5A–E; Takagi et al., 2017). Moreover, the density of microglia and macrophages is more significant in the CVOs than in other brain regions (Figure 5F; Takagi et al., 2017).

In addition to the amoeboid shape, microglia and macrophages in the CVOs express higher levels of M1 marker proteins CD16/32 and CD86 and M2 marker proteins CD206 and Ym1 than other brain regions (Figures 5G–I; Takagi et al., 2017). Peripheral macrophages are classified into two activated states; the M1 phenotype, which shows a classical proinflammatory response by the production of inflammatory cytokines and reactive oxygen species, antigen presentation, and the removal of pathogens (Murray, 2017), and the M2 phenotype that expresses mediators or receptors to down-regulate, repair, or protect the body from inflammation (Mantovani et al., 2004). M1/M2 classification of macrophages has also been applied to microglia in the brain, although the M1/M2 categories have limitations (Randsohoff, 2016). This demonstrates that microglia and macrophages in the CVOs are continuously activated, considering the amoeboid morphology and M1 and M2 protein expression.

The reason for continuous activation of microglia and macrophage is not well understood. Endothelial cells in the CVOs lack tight junction proteins, allowing blood-derived molecules into the parenchyma (Morita and Miyata, 2012, 2013; Morita et al., 2016). CD163-expressing macrophages in AP perivascular space sequester dextran 10 kDa (molecular weight 10,000) within 5 min of administration (Willis et al., 2007). Perivascular macrophages in the pineal gland express M1 marker, MHC II and display phagocytosis activity (Kaur et al., 1997; Møller et al., 2006). In considering the function of these M1 and M2 marker proteins, activated microglia and macrophages phagocytize neurotoxic blood-derived molecules or cells to maintain CVO parenchyma microenvironment (Wang et al., 2014). Another possible activated microglia role is in structural dynamics such as neurogenesis and angiogenesis. Continuous angiogenesis with proliferation and apoptosis of endothelial cells occurs in the CVOs of adult mice (Morita et al., 2013, 2015; Furube et al., 2014; Asami et al., 2020). Conditioned media of resting-state microglia inhibits brain endothelial cell proliferation, whereas that from the activated-state microglia facilitates proliferation (Welser et al., 2010). Activated microglia increase vascular branching density in the retina (Biswas et al., 2017). Thus, activated microglia may regulate endothelial cell proliferation or removal of apoptotic endothelial cells. Activated microglia in the subventricular zone of developing brains promote neurogenesis and oligodendrogenesis (Shigemoto-Mogami et al., 2014). Differentiation of NPCs with M1 microglia supernatant leads to neurogenesis, while exposure to M2 supernatants leads to oligodendrogenesis (Butovsky et al., 2006; Yuan et al., 2017). Activated microglia are likely involved in controlling of oligodendrogenesis and neurogenesis by acting on NSCs and/or their progenitor cells.

### Inflammation-induced microglial proliferation in the brain

**FIGURE 6:**
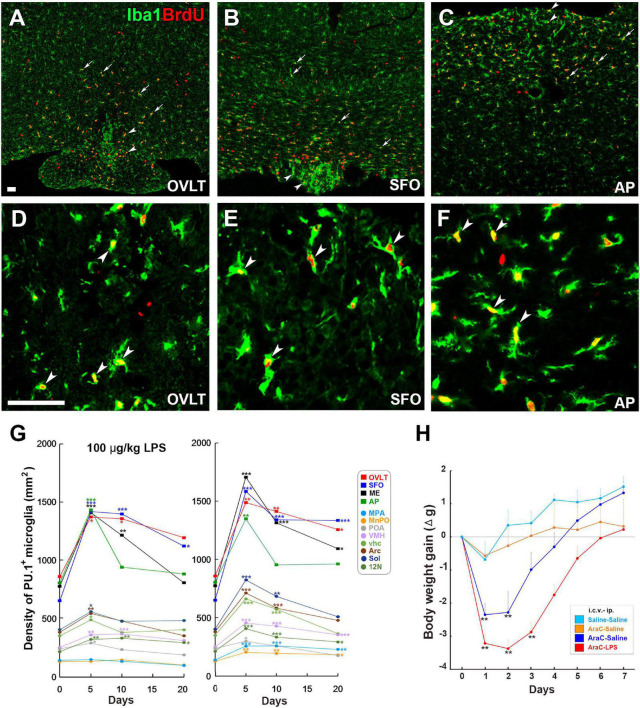
Remarkable increase in proliferation of microglia and macrophages in the CVOs and neighboring brain regions in adult mouse brain after LPS administration. Intraperitoneal administration of 100 μg/kg LPS induces a robust increase of Iba1^+^ BrdU^+^ proliferating microglia and macrophages in the OVLT (arrowheads) and medial preoptic area (MPA; arrows) **(A)**, the SFO (arrowheads) and ventral hippocampal commissure (vhc; arrows) **(B)**, and the AP (arrowheads) and solitary nucleus (Sol; arrows) **(C)**. High magnification reveals many Iba1^+^ BrdU^+^ proliferating microglia and macrophages in the CVOs **(D–F)**. Scale bars = 100 **(A)** and 50 μm **(D)**. Photographs are rearranged with permission from Springer Nature (Furube et al., 2015, 2018). The density of PU.1^+^ microglia and macrophages is significantly elevated in the CVOs and neighboring brain regions on the 5th and 10th day after administration of 100 μg/kg and 1 mg/kg LPS, but returns to almost normal levels on the 20th day **(G)**. The i.c.v. infusion of the mitotic inhibitor AraC significantly delays body weight recovery after 5 mg/kg administration **(H)**. 10N, dorsal motor nucleus of vagus nerve; 12N, hypoglossal nucleus; AP, area postrema; fi, fimbria; MnPO, median preoptic area; MPA, medial preoptic area; OVLT, organum vasculosum of the lamina terminalis; SFO, subfornical organ; Sol, solitary nucleus; vhc, ventral hippocampal commissure; VMH, ventromedial hypothalamic nucleus. *p < 0.05, **p < 0.01, ***p 0.001 vs. the control by an ANOVA with Tukey’s *post-hoc* test. Graphs are rearranged with permission from Springer Nature [**(A)** Furube et al., 2018] and Elsevier B.V. [**(B)** Torii et al., 2022].

Microglia are long-lived cells in adult mammalian brains, and their density remains stable under normal physiological conditions, although their turnover occurs several times in a lifetime through the spatial and temporal coordination of proliferation and apoptosis (Askew et al., 2017). Robust microglial proliferation often occurs under severe pathological brain conditions such as stroke-induced brain injury (Hou et al., 2020), spinal cord injury (Bellver-Landete et al., 2019), traumatic brain injury (Henry et al., 2020), and Alzheimer’s disease (Olmos-Alonso et al., 2016). Recently, we revealed that LPS-induced microglial proliferation occurs in brain regions, such as the hypothalamus, medulla oblongata, and telencephalon, with marked increases in the CVOs, by LPS administration at a low dose of 100 μg/kg (Figures 6A–F; Furube et al., 2018). Moreover, the single peripheral administration of 1 mg/kg LPS induced robust microglial proliferation in most brain regions, except for the cerebral cortex and hippocampus, in the adult mouse (Fukushima et al., 2015; Furube et al., 2015, 2018). The CVOs are the blood-brain interface to initiate early stages of brain inflammation; prominent activation of STAT3 (Rummel et al., 2005; Yoshida et al., 2016) and NF-κB signaling (Muneoka et al., 2019), and increased expression of Fos (Brochu et al., 1999) and cytokine mRNA (Quan et al., 1997; Brochu et al., 1999). On the other hand, a proliferation of microglia does not occur in the cerebral cortex and hippocampus after peripheral LPS administration of 100 and 1 mg/kg. This indicates increased proliferation of microglia during LPS-induced inflammation in a region- and dose-dependent manner, demonstrating proliferation sensitivity variations of microglia. Microglial proliferation peaks 24–72 h after intraperitoneal LPS injection accompanied by increased microglial density, but the density returns to normal levels within 3 weeks (Figure 6G; Furube et al., 2018). Microglial proliferation is also observed during inflammation induced by the toll-like receptor 2 (TLR2) agonist zymosan and PGE_2_ (Torii et al., 2022). These results indicate that microglial proliferation is a general event of neuroinflammation rather than a specialized one in severe neurodegenerative diseases accompanied by neuronal death.

The inhibition of LPS-induced microglial proliferation with continuous i.c.v. infusion of AraC causes a persistent reduction in body weight and intake of food and water and prolongs LPS-induced sickness responses, such as lower locomotor activity and core body temperature (Figure 6H; Torii et al., 2022). Thus, a transient increase in the microglial population is beneficial during endotoxin-induced inflammation as it attenuates sickness response. The selective elimination of microglia with a colony-stimulating factor_1_ receptor (CSF_1_R) antagonist increased neuronal death in an acute stroke model (Hou et al., 2020) and spinal cord injury (Bellver-Landete et al., 2019). In contrast to the neuroprotective function in acute injury, the elimination of microglia with a CSF_1_R antagonist attenuates neurodegeneration during the chronic phase of traumatic brain injury (Henry et al., 2020) and Alzheimer’s (Olmos-Alonso et al., 2016). Therefore, microglia appear to exert beneficial or deleterious effects depending on brain disease, beneficial in acute inflammation and deleterious in chronic inflammation.

### Fluid homeostasis by osmolality and Na^+^ sensitive sensor in the circumventricular organs

**FIGURE 7:**
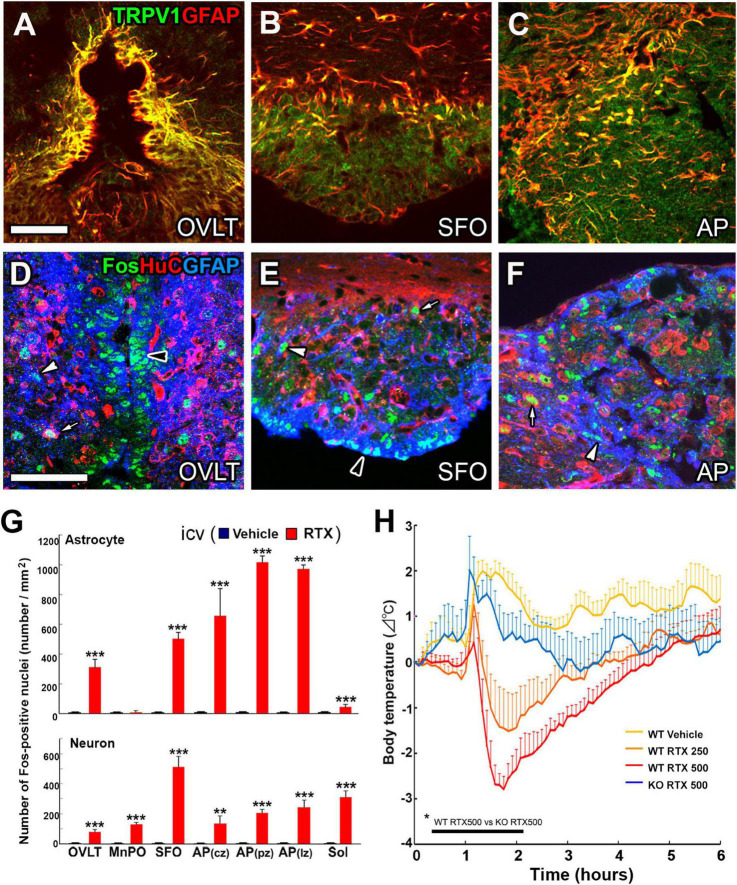
Prominent TRPV1 and TRPV1-dependent Fos expression at astrocytes in the CVOs of adult mouse brain and hypothermia by central activation of TRPV1. The expression of TRPV1 is prominent at GFAP^+^ astrocytes in the CVOs **(A–C)**. The i.c.v. infusion of the TRPV1 agonist resiniferatoxin at 500 ng/kg causes prominent Fos expression at astrocytes rather than neurons in the CVOs **(D–F)**. AP, area postrema; GFAP, glial fibrillar acidic protein; OVLT, organum vasculosum of the lamina terminalis; SFO, subfornical organ. Scale bars = 50 μm. Quantitative analysis shows central infusion of resiniferatoxin induces significant Fos expression at astrocytes preferentially in the CVOs and at neurons in the CVOs and neighboring brain regions **(G)**. The i.c.v. infusion of the TRPV1 agonist resiniferatoxin induces hypothermia in a dose-dependent manner in WT mice, but not in TRPV1 KO mice **(H)**. **p < 0.01, ***p0.001 between WT and TRPV1 KO mice with unpaired Student’s *t*-test **(G)** or an ANOVA with Tukey’s *post-hoc*-test **(H)**. Photographs and graphs are rearranged with permission from John Wiley & Sons [**(A–G)** Mannari et al., 2013] and Springer Nature [**(H)** Yoshida et al., 2016].

Animals with the electrolytic lesion of the OVLT and SFO, namely AV3V region, show a decreased response to dehydration or hypertonic saline ingestion, exhibiting reduced fluid intake, Fos expression, and AVP and OT secretion (Morris et al., 1994; Miyata et al., 1996; Rocha et al., 1999; Oliveira et al., 2004), indicating that AV3V region is crucial for the reception and integration of osmotic signals. TRPV1 is the first in the thermosensitive TRP family of proteins or non-selective cation channels (Caterina et al., 1997; Julius, 2013). TRPV1 is activated by noxious high temperatures (>42°C), pH, positive voltages, and chemicals such as capsaicin (Julius, 2013). Moreover, TRPV1 is involved in mechanical pain and high osmolality detection by responding to cellular shape alterations (Bourque, 2008). Although TRPV1 is known to be expressed at somatosensory neuron nerve endings (Romanovsky et al., 2009), it is also highly expressed in adult brain CVOs (Ciura and Bourque, 2006; Mannari et al., 2013; Sladek and Johnson, 2013). Moreover, prominent expression of TRPV1 is detected at astrocytic and tanycytic cellular processes, which contact closely with fenestrated capillaries and form dense networks (Figures 7A–C; Mannari et al., 2013). The TRPV1 deficiency of the OVLT results in decreased sensitivity to hyperosmolality (Ciura and Bourque, 2006). Full-length TRPV1 responds to hyperosmolality but is less sensitive to hypoosmolality (Nishihara et al., 2011). In contrast to these *in vitro* experiments, acute and chronic hyperosmolality produces a similar increase in the number of Fos-positive neurons in the OVLT, AVP secretion, and water intake in WT vs. TRPV1 KO mice (Taylor et al., 2008; Kinsman et al., 2014; Tucker and Stocker, 2016). These results indicate that TRPV1 is not responsible for *in vivo* body fluid homeostasis.

An alternative candidate for *in vivo* fluid homeostasis control is the Na_*x*_ channel, which detects Na^+^ levels (Noda, 2006, 2007; Noda and Hiyama, 2015). The expression of the Na_*x*_ channel occurs specifically at astrocytes and tanycytes in the CVOs such as the OVLT, SFO, ME, and NH (Watanabe et al., 2000, 2006). The Na_*x*_ channels are activated depending on Na^+^ concentration, but not osmolality, with a threshold of about 150 mM (Hiyama et al., 2002). WT mice show extensive water intake and aversion to saline intake, whereas Na_*x*_ KO animals did not exhibit such behaviors (Hiyama et al., 2004). Abnormal behaviors, such as water intake and aversion to saline in Na_*x*_ KO mice, are recovered by a site-directed infection of an adenoviral vector with the Na_*x*_ gene into the SFO, indicating that the SFO is the primary brain center regulating water and salt intake behaviors (Hiyama et al., 2004).

### Warm-sensitive transient receptor potential vanilloid 1-expressing glial cells in the circumventricular organs

Body thermoregulation is closely connected to body water homeostasis, and the CVOs act as integrators of thermal and osmotic signals in the brain (Sladek and Johnson, 2013). The physiological benefits of this are; first, the CVOs lack the typical BBB to protect from blood-derived molecules such as ions and low and high molecular hydrophilic molecules (Morita and Miyata, 2012; Miyata, 2015), but they directly and rapidly detect ions, osmolality, and blood temperature. Second, the capillary density is higher in the CVOs than in other brain regions, enabling efficient detection of blood-derived signal. The peripheral and central administration of the TRPV1 agonist resiniferatoxin, induced Fos expression preferentially in the CVO astrocytes and tanycytes and neurons of the median preoptic nuclei and Sol (Figures 7D–F; Mannari et al., 2013). Moreover, the peripheral and central administration of resiniferatoxin robustly induces STAT3 signaling in astrocytes of the CVOs and POA, but less in neurons (Figure 7G; Mannari et al., 2013). A similar response to the peripheral and central administration of LPS (Rummel et al., 2004, 2005; Nakano et al., 2015). IL-6 activates STAT3 signaling in the brain (Akira, 1997) and is necessary to induce fever (Chai et al., 1996; Nilsberth et al., 2009). The activation of TRPV1 stimulates c-Jun and p38 mitogen-activated protein kinases, releasing cytokines IL-6 and -8 in CD4^+^ cells (Bertin et al., 2014). The central administration of RTX causes a strong dose-dependent decrease in body temperature (Figure 7H; Yoshida et al., 2016). Peripheral administration of LPS induces less fever response in TRPV1 KO mice compared with WT animals (Iida et al., 2005). Thus, TRPV1 is necessary to maintain LPS-induced fever, as LPS sensitizes TRPV1 through TLR4 activation in the trigeminal sensory neurons (Diogenes et al., 2011).

**FIGURE 8:**
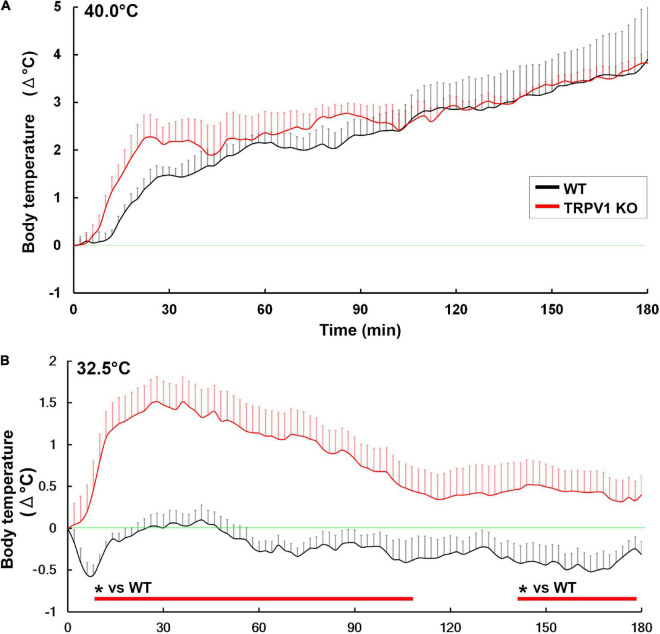
Abnormal hyperthermia in TRPV1 KO mice upon exposure to moderately warm ambient temperature. Both WT and TRPV1 KO mice showed similar increases in core body temperature upon exposure to 40°C **(A)**. Upon exposure to 32.5°C, TRPV1 KO mice exhibited hyperthermia, but hyperthermia was not seen in WT mice **(B)**. *p < 0.05 between WT and TRPV1 KO mice with unpaired Student’s *t*-test. Graphs are rearranged with permission from Springer Nature (Park et al., 2020).

The deletion of TRPV1-expressing somatosensory neurons eliminates heat avoidance behavior in two-plate preference tests (Pogorzala et al., 2013). However, TRPV1 KO mice are dispensable for typical heat avoidance between 40 and 50°C by two-plate preference tests (Pogorzala et al., 2013) and normal warm sensation between 32 and 42°C by goal-directed thermal perception task (Paricio-Montesinos et al., 2020). No significant difference is detected in colonic temperature between WT and TRPV1 KO mice when exposed to 37.0°C (Szelenyi et al., 2004), 39.0°C (Garami et al., 2011), or 40°C (Figure 8A; Park et al., 2020). This raises the question as to the function of TRPV1 upon acute ambient temperature increase. Recently, our research suggests that TRPV1 is crucial for thermoregulation by initiating heat loss behaviors under warm ambient temperatures less than 37°C (Figure 8B; Park et al., 2020). TRPV1 KO mice showed significantly shorter durations for heat loss behaviors such as sleeping and body licking than WT mice upon exposure conditions between 30.0 and 35.0°C (Park et al., 2020). On the other hand, no significant difference in heat loss behavior time is observed between WT and TRPV1 KO mice upon exposure to 40°C (Park et al., 2020), indicating that exposure to heat greater than body temperature is beyond the cooling capacity of these heat loss behaviors. Thus, TRPV1 is necessary for thermoregulation under moderate warm ambient temperatures to control heat loss behaviors. The oral gavage of the TRPV1 agonist capsaicin quickly decreases core body temperature together with increased tail surface temperature in WT mice *via* activation of the CVOs and POA, whereas TRPV1 KO animals do not exhibit such changes (Inagaki et al., 2019). Moreover, oral gavage of capsaicin induces a long-lasting increase in locomotor activity in a TRPV1-independent manner, as increased locomotor activity was seen in both WT and TRPV1 KO animals (Inagaki et al., 2019). Capsaicin dilates blood vessels in the skin, and increases heat exchange and metabolic activity in humans (Luo et al., 2011; Reyes-Escogido Mde et al., 2011; Adaszek et al., 2019).

Transient receptor potential vanilloid 1 is expressed in astrocytes and tanycytes of the CVOs, as previously mentioned (Mannari et al., 2013; Sladek and Johnson, 2013). Central and peripheral administration of RTX causes STAT3 signaling activation and Fos expression at astrocytes and tanycytes rather than neurons in the CVOs (Mannari et al., 2013). In the CVOs, the fenestrated capillaries are surrounded by dense networks of astrocytic and tanycytic cellular processes. Neuronal somata are generally located at a moderate distance from fenestrated capillaries and are covered with glial cellular processes (Miyata, 2015; Morita et al., 2016). This unique spatial niche is necessary to protect neurons from molecules and ions that diffuse beyond the fenestrated capillaries. Hypoosmolality results in an influx of water into the brain parenchyma *via* glial cells, then brain water is shunted into astrocytes, which preserves neuronal cell size and prevents brain damage (Ayus et al., 2008). These results indicate that TRPV1-expressing astrocytes and tanycytes are “warm-sensitive glial cells.”

### Glia-neuronal communication in the circumventricular organs and transmission to other brain regions

Bidirectional communications between glial cells and neurons are essential for axonal conduction and synaptic transmission (Fields and Stevens-Graham, 2002). A transient increase in astrocytic Ca2^+^ levels results in a gliotransmitter release, which acts on neurons and vascular smooth muscle (Bazargani and Attwell, 2016). Raising the question of how the fluid or thermal information sensed by astrocytes and tanycytes is then transferred to neurons. Coordinated activation of Na_x_ and Na^+^/K^+^ ATPase enhances glucose uptake and extensive lactate production in astrocytes and tanycytes in the SFO in response to elevated Na^+^ levels (Shimizu et al., 2007). Moreover, the Na^+^-dependent release of the glial transmitter lactate from astrocytes and tanycytes stimulates GABAergic neurons in the SFO to induce water intake behavior (Shimizu et al., 2007). Endothelin-3 decreases the Na_x_ sensitivity threshold of Na^+^ from ∼ 150 to 135–145 mM and increases lactate release in the SFO (Hiyama et al., 2013). Thus, thermal information detected by TRPV1 in astrocytes and tanycytes is also transferred to neurons by glial transmitters.

Peripheral administration of a TRPV1 agonist induces hypothermia (Caterina et al., 1997), but a TRPV1 antagonist causes fever (Szelenyi et al., 2004). While central administration of TRPV1 agonists induced hypothermia, TRPV1 antagonists had no effect (Yoshida et al., 2016; Park et al., 2020). These results indicate that peripheral TRPV1 is always activated, but central TRPV1 is inactivated. The glial-neuronal pathways of activated TRPV1 in the CVOs are well understood; however, the hypothermic brain center was recently reported. Opsin 5-, adcyap1-, or QRFP-expressing neurons in the lateral hypothalamic area around the third ventricle of the anterior part of the hypothalamus are shown to be hypothermic and hypometabolic neurons (Hrvatin et al., 2020; Takahashi et al., 2020; Zhang K. X. et al., 2020). These hypothalamic neurons are closely located to the OVLT, indicating that glial TRPV1 activation information is transferred to these neurons, thereby causing hypothermia.

Thermal and osmotic information monitoring and integration in the OVLT and SFO are sent to the median preoptic area (MnPO) that functions as osmoregulation, thermoregulation, and sleep homeostasis (Sladek and Johnson, 2013). In addition, the OVLT and SFO project to the supraoptic and paraventricular nuclei, which are the autonomic center, oxytocin, and vasopressin-synthesizing regions (Miyata and Hatton, 2002; Sladek and Johnson, 2013). The SFO and OVLT respond to blood angiotensin II, relaxin, and hyperosmolarity to drive thirst-related neural pathways. However, amylin and leptin act at the AP to influence neural pathways inhibiting food intake (McKinley et al., 2019). Activation of ADCYAP1-expressing neurons in the AP and Sol induces LPS-induced sickness responses such as reduced food and water intake, locomotor activity, and body temperature using the TRAP2 system. In contrast, inhibition of these neurons significantly weakens all responses except for effect on body temperature (Ilanges et al., 2022). Moreover, the activation of PHOX2B-expressing neurons in nearly all AP cells and some NTS cells and DBH-expressing neurons in LPS-activated AP neurons decreases food and water intake and locomotor activity (Ilanges et al., 2022). These data indicate that ADCYAP1-expressing neurons in the AP and Sol are essential for endotoxin-induced sickness behaviors.

## Author contributions

The author confirms being the sole contributor of this work and has approved it for publication.
